# The Association Between Three *MMP3* Gene Polymorphisms and the Efficacy of Platelet-Rich Plasma Therapy in the Treatment of Lateral Elbow Tendinopathy—A Prospective Cohort Study

**DOI:** 10.3390/ijms262110579

**Published:** 2025-10-30

**Authors:** Alicja Jarosz, Tomasz Nowak, Justyna Wrona, Anna Balcerzyk-Matić, Tomasz Iwanicki, Karol Szyluk, Joanna Iwanicka, Wojciech Kania, Katarzyna Gawron, Paweł Niemiec

**Affiliations:** 1 Department of Biochemistry and Medical Genetics, Faculty of Health Sciences in Katowice, Medical University of Silesia in Katowice, Medykow 18 Str., 40-752 Katowice, Poland; tnowak@sum.edu.pl (T.N.); justyna.wrona@sum.edu.pl (J.W.); abalcerzyk@sum.edu.pl (A.B.-M.); tiwanicki@sum.edu.pl (T.I.); jiwanicka@sum.edu.pl (J.I.); 2 District Hospital of Orthopaedics and Trauma Surgery, Bytomska 62 Str., 41-940 Piekary Slaskie, Poland; kszyluk@o2.pl; 3 Department of Physiotherapy, Faculty of Health Sciences in Katowice, Medical University of Silesia in Katowice, Medykow 12 Str., 40-752 Katowice, Poland; 4 Department of Trauma and Orthopedic Surgery, Multidisciplinary Hospital in Jaworzno, Chelmonskiego 28 Str., 43-600 Jaworzno, Poland; wojtekkania@poczta.onet.pl; 5 Department of Medical Microbiology, Faculty of Medical Sciences in Katowice, Medical University of Silesia, Medykow 18, 40-752 Katowice, Poland; kgawron@sum.edu.pl

**Keywords:** PRP, MMP3, SNP, platelet-rich plasma, matrix metalloproteinase 3, lateral elbow tendinopathy

## Abstract

Matrix metalloproteinases (MMPs) are proteolytic enzymes involved in connective tissue remodeling. Matrix metalloproteinase 3 (MMP3) belongs to the MMP family and is associated with the pathogenesis of tendinopathy. Moreover, *MMP3* gene polymorphisms have been associated with the risk of tendinopathy development. The goal of this study was to investigate whether this gene polymorphisms could also affect the effectiveness of platelet-rich plasma (PRP) tendinopathy treatment. 107 patients (132 elbows) with lateral elbow tendinopathy underwent PRP injection and were followed for two years at specific follow-up weeks (2, 4, 8, 12, 24, 52, 104). The effectiveness of the therapy was assessed based on patient-reported outcome measures (PROMs) values, specifically visual analogue scale (VAS), quick version of the disabilities of the arm, shoulder and hand (QDASH), patient-rated tennis elbow evaluation (PRTEE), and the achievement of minimal clinically important difference (MCID). Three *MMP3* single nucleotide polymorphisms (SNPs) (rs520540, rs591058, rs679620) were genotyped using the TaqMan method. All studied polymorphisms were found to present strong linkage disequilibrium and were associated with the effectiveness of therapy on the VAS scale (week 4) and PRTEE (week 104), as well as with MCID achievement (PRTEE week 4); however, these were not strong associations. The studied SNPs also showed an association with the frequency of hand pain before treatment. *MMP3* gene polymorphisms are associated with pain experienced before PRP therapy, but do not show a clear association with treatment effectiveness.

## 1. Introduction

Matrix metalloproteinases (MMPs) belong to multidomain zinc-dependent endopeptidases, i.e., the metzincin protease superfamily [1]. Humans produce 23 MMPs, which are mainly responsible for extracellular matrix (ECM) remodeling [2,3]. Due to their function, MMPs control many essential processes, such as transcriptional control, cell signaling, immune regulation [3], embryonal development, ovulation, wound healing, periodontitis, tumor invasion, and metastasis, as well as soft tissue remodeling after injury [4]. Therefore, dysregulation in MMPs activity may lead to diseases such as fibrosis, cancer, arthritis, chronic ulcers, nephritis, encephalomyelitis, and others [2]. This shows how important MMPs are in both the physiology and pathology of human tissues. The activity of MMPs is regulated by tissue inhibitors of metalloproteinases (TIMPs) [2]. All TIMPs inhibit MMPs through reversible blockade. They act by selectively inhibiting specific MMPs, as well as by activating and removing them from the ECM [5]. Balance between TIMPs and MMPs activity is crucial for proper ECM remodeling.

Due to MMPs’ role in the degradation of connective tissue components and ECM modification, they are also involved in the development of tendinopathy [4]. Tendinopathy is defined as a clinical syndrome characterized by pain, swelling, and impairment in and around tendons. The molecular mechanism of tendinopathy includes, among others, disturbances in ECM structure, tenocytes proliferation and angiogenesis [6]. The etiology of tendinopathy remains unclear; current hypotheses include hypoxia, ischemic injury, oxidative stress, or MMP imbalance [7]. Indeed, comparative studies of healthy and diseased tendons have shown differences in the activity of many proteins from the MMP family [4]. These differences may be related to both the pathogenesis and healing of the injury. For example, studies on rotator cuff tears indicate an increase in MMP13 levels, which is an expression of active tissue remodeling. However, it is unknown whether these changes are a healing mechanism or a secondary effect of the tear itself [8]. It has been shown that MMPs may predispose to painful tendinopathy and tendon rupture [9] and their activity leads to collagen damage [10]. However, degradation is crucial for the healing and remodeling of connective tissue. In tendinopathy, ECM disorganization occurs due to an imbalance in the type I to type III collagen ratio. After tendon damage, type III collagen is synthesized first, and is replaced by type I collagen under physiological conditions. However, during tendinopathy, an increased proportion of type III to type I collagen is observed, which leads to the persistence of disease symptoms [11]. Therefore, collagen degradation and a balance of MMP activity are essential for tendon regeneration.

Matrix metalloproteinase 3 (MMP3) also known as stromelysin 1 is encoded by the gene of the same name (*MMP3*, location 11q22.2) [12]. As a member of the MMPs, the MMP3 enzyme degrades different proteins, including proteoglycans, fibronectin, elastin, laminin, and collagen (II, III, IV, IX and X types). Importantly, MMP3 also influences the activity of other metalloproteinases, such as MMP1, MMP7, and MMP9 [13]. All this demonstrates the significant function of MMP3 in tissue remodeling. MMP3 is also associated with tendinopathy. *MMP3* is expressed in healthy tendons [9]. However, its levels are significantly reduced in cases of painful tendinopathy or Achilles tendon rupture [14]. A decrease in *MMP3* expression has also been shown in torn rotator cuff tendons [8]. Moreover, several single nucleotide polymorphisms (SNPs) of the *MMP3* gene showed association with the risk of tendinopathy, including rs679620 and rs591058 [15,16,17].

The aim of the current study was to analyze the association between *MMP3* gene SNPs and the effectiveness of lateral elbow tendinopathy (LET) treatment using platelet-rich plasma (PRP). LET is a tendinopathy of the forearm extensor muscles, often caused by overuse, repetitive use, or direct trauma to the epicondyle [18]. While PRP is a form of tendinopathy treatment that uses high concentration of platelets and their growth factors for accelerating tissue regeneration [19,20,21,22]. However, PRP’s effectiveness in tendinopathy treatment is widely debated in the literature [23]. We believe that individual variability in response to PRP therapy may be due to genetic variation. Therefore, we decided to investigate whether polymorphisms associated with the pathogenesis of tendinopathy would also modify the effectiveness of its treatment with PRP. We selected the *MMP3* gene for this study due to numerous associations of its polymorphisms with the risk of tendinopathy [15,16,17] and changes in its expression during tendon damage [8,14]. Three *MMP3* polymorphisms were selected: rs679620 and rs591058, which have been previously associated with the risk of tendinopathy, and rs520540, which has not been analyzed in this context. The rs520540 SNP was included in our research mainly due to its frequency in the studied population.

## 2. Results

### 2.1. Haplotype Analysis and Polymorphisms Characteristics

**Figure 1:**
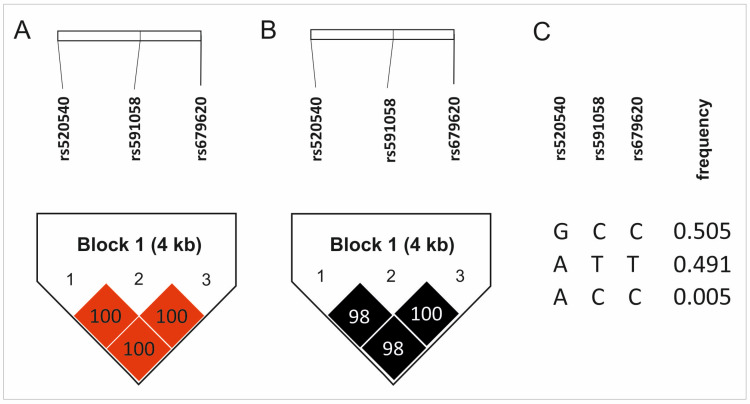
Results of haplotype analyses: (**A**) D’ values, (**B**) R^2^ values, (**C**) haplotype frequency. Color intensity represents the magnitude of linkage disequilibrium.

Three *MMP3* gene polymorphisms were analyzed (rs520540, rs591058, rs679620). The distribution of alleles and genotypes for all of them were consistent with Hardy–Weinberg equilibrium (HWE). The rs520540 is a synonymous variant, rs591058 is an intron variant, while rs679620 is a missense variant with a benign clinical significance [24]. The genotypes and allele frequencies of the studied SNPs, along with their chromosomal locations and the results of HWE analyses, are presented in Table 1. The studied polymorphisms showed very strong linkage disequilibrium, even though they are located at a relatively substantial distance from each other (Figure 1). For this reason, the results for all analyzed polymorphisms almost completely overlap, SNPs rs591058 and rs679620 show identical distributions.

### 2.2. Association Between the Studied Polymorphisms and PROMs Value

**Figure 2:**
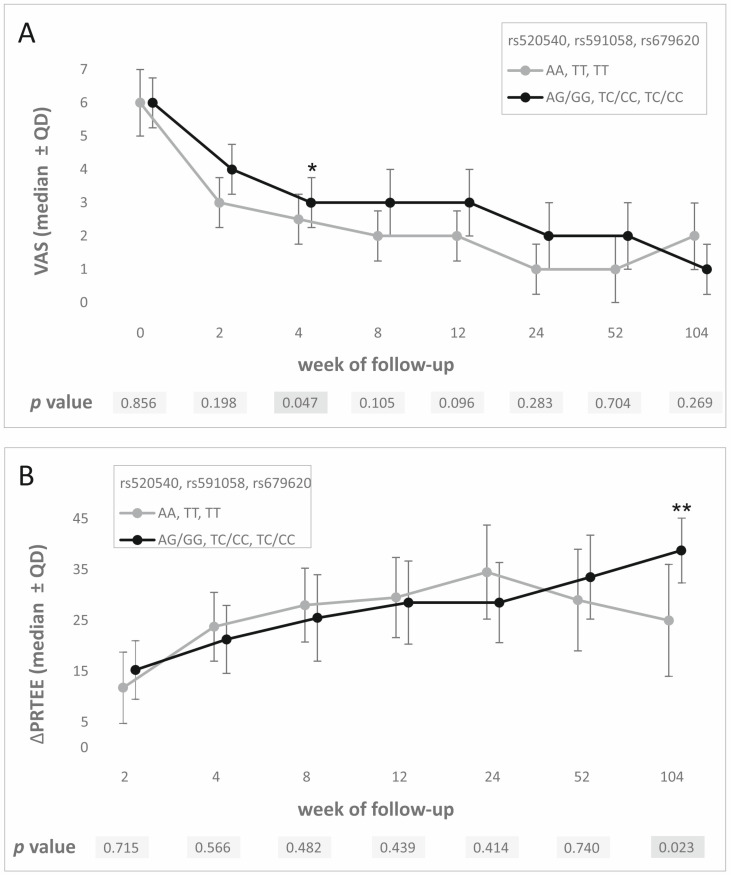
PROM analysis results—medians ± QD at specific follow-up weeks for all polymorphisms studied for (**A**) VAS, (**B**) ΔPRTEE. Legend: VAS, visual analogue scale; PRTEE, patient-rated tennis elbow evaluation; QD, quartile deviation; *, statistically significant results; **, statistically significant results after Hochberg correction.

A strong linkage disequilibrium of studied SNPs was reflected in PROMs analysis results. Differences at the same follow-up weeks were observed for all three polymorphisms. VAS values at week 4 were significantly lower for AA homozygotes (vs. GG/AG; rs52540, *d* = 0.33), TT homozygotes (vs. CC/TC; rs591058, *d* = 0.33), and TT homozygotes (vs. CC/TC; rs679620, *d* = 0.33). These results were not statistically significant after the Hochberg correction. However, carriers of G (rs52540), C (rs591058), and C (rs679620) alleles showed a better response to PRP therapy on the PRTEE scale (higher ΔPRTEE, week 104 of follow-up, *d* = 0.67 for all SNPs). These results were statistically significant after the Hochberg correction (*p* value assessed as 0.023) (Figure 2). Detailed results of PROM analyses for all studied polymorphisms are presented in Tables S1–S3. Moreover, homozygote GG of rs520540, CC of rs679620, and CC of rs591058 achieved MCID more frequently than carriers of other genotypes. Similar results were obtained for allelic associations (G, C, and C alleles respectively) (Table 2). All results for MCID analysis were statistically important after Hochberg correction (*p* value assessed as 0.007). Due to the strong linkage disequilibrium between the studied SNPs, we also analyzed the influence of the GCC haplotype (the most common in the study group) on PROM values. Carriers of this haplotype showed higher VAS values at week 4 (median ± QD: 3.0 ± 1.5 vs. 2.5 ± 1.5, *p* = 0.005), higher ΔPRTEE values at week 104 (median ± QD: 38.75 ± 12.75 vs. 25.00 ± 22.00, *p* = 0.001), and lower ΔPRTEE values at week 24 (median ± QD: 28.50 ± 15.75 vs. 34.50 ± 18.50, *p* = 0.001).

### 2.3. MMP-3 Gene Polymorphisms and Pain Characteristics

**Figure 3:**
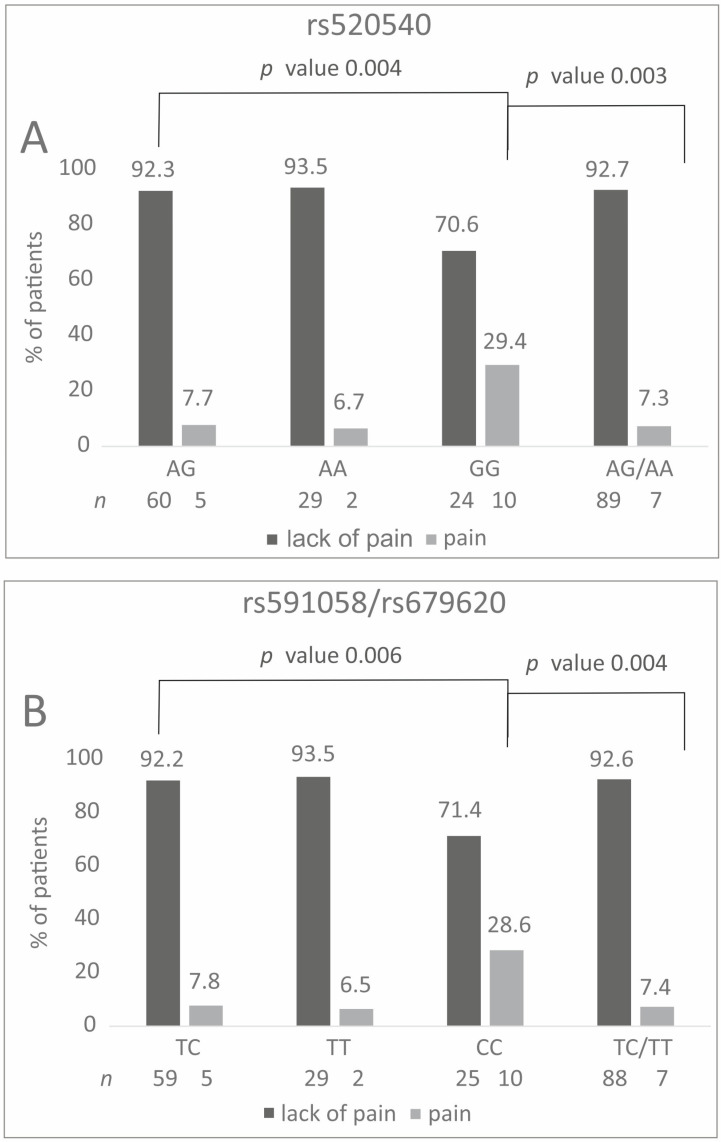
Percentage distribution of specific genotypes carriers in relation to the experience of hand pain before therapy. Results for (**A**) rs520540, (**B**) rs591058 and rs679620.

All analyzed polymorphisms showed an association with pain characteristics. Patients with the GG genotype of rs520540 experienced hand pain more frequently than carriers of the A allele (29.4% vs. 7.3%, *p* = 0.003). Hand pain was also reported more often by CC homozygotes than by carriers of the T allele for the rs591058 and rs679620 polymorphisms (28.6% vs. 7.4%) (Figure 3). Statistically significant differences were also observed in the additive model for each polymorphism (Figure 3, Table S4). All differences remained statistically significant after Hochberg correction (*p* value was assessed as 0.006).

### 2.4. In Silico Expression Analyses

**Figure 4:**
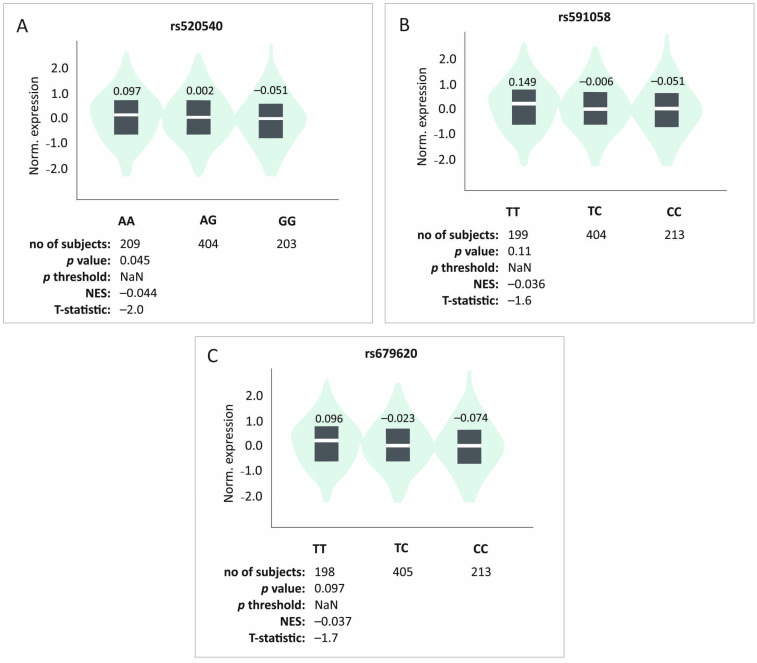
In silico analyses of the studied polymorphisms’ effect on the level of *MMP3* gene expression in musculoskeletal tissues. Results for (**A**) rs520540, (**B**) rs591058, (**C**) rs679620. Made using GTEx tool [25].

In silico analyses of the studied SNPs effect on *MMP3* gene expression levels in musculoskeletal tissues were not statistically significant, but they did indicate a trend (rs520540 GTEx *n* = 816, *p* = 0.045, *p* threshold = NaN; rs591058 GTEx *n* = 813, *p* = 0.11, *p* threshold = NaN; rs679620 GTEx *n* = 816, *p* = 0.097, *p* threshold = NaN). The G, C, and C alleles (rs520540, rs591058, and rs679620, respectively) that showed strong linkage disequilibrium and create the most common haplotype were associated with lowest expression levels (Figure 4).

## 3. Discussion

The current study analyzed the associations between *MMP3* gene polymorphisms and the effectiveness of PRP therapy in the treatment of lateral elbow tendinopathy. The results for the studied SNPs almost completely overlap, which stems from their strong linkage disequilibrium. In the case of PROMs and MCID analyses, we obtained conflicting results. The AA, TT, and TT (rs520540, rs591058, and rs679620) genotypes were associated with greater treatment progress on the VAS scale (week 4), whereas carriers of the G, C, and C alleles (rs520540, rs591058, and rs679620) demonstrated more effective treatment on the PRTEE scale (week 104). The same alleles (G, C, and C) were also associated with greater treatment effectiveness in MCID analyses. MCID is used to determine whether therapy had a clinically significant effect [26]. The results for the GCC haplotype’s association with PROM values were consistent with the results for single SNP analyses, except for PRTEE week 24. Importantly, only the results from the VAS analyses were not statistically significant after applying the correction for multiple testing. This suggests that the results for the PRTEE and MCID analyses are significant, meaning that the G, C, and C alleles may be associated with better PRP therapy efficacy. The differences in the results obtained for VAS (week 4) and PRTEE (week 104) may be due to the healing phases. The tendon healing process can be divided into three phases: inflammatory, proliferative, and remodeling [27,28]. The inflammatory phase initiates the response to injury within the first week [27]. During this phase, vascular permeability increases and inflammatory cells influx into the healing site [28]. This is followed by a proliferation phase within 1–4 weeks, during which cells proliferate and collagen is produced, although it is not well organized [27]. Finally, after 4 weeks, the remodeling phase starts, in which tissue remodeling and maturation occur, including the replacement of type III collagen with type I collagen [29]. Therefore, MMPs appear to be particularly important during this period. Interestingly, week 4 is between the proliferation and remodeling phases, which may influence MMP function and the results obtained. Furthermore, in the case of MMP3, studies in animal models have shown that this protein is likely associated with both collagen degradation and remodeling, which is consistent with our results [30]. However, based on the obtained results, it should be emphasized that the relationship between *MMP3* gene polymorphisms and the effectiveness of PRP therapy seems to be questionable. The lack of association between *MMP3* gene polymorphisms and the effectiveness of PRP therapy is evidenced by the lack of significant differences in PROMs values between individual genotypic variants during the course of treatment (out of 45 observation points for each polymorphism, statistically significant differences are visible only in two points; Tables S1–S3). Despite this, we were able to demonstrate a relationship between the studied SNPs and the pain experienced by patients before treatment. Carriers of the GG (rs520540), CC (rs591058), and CC (rs679620) genotypes experienced hand pain more frequently than other patients. Finally, the same genotypes (GG, CC, and CC) showed a trend according to which they are associated with reduced *MMP3* gene expression.

Two of the analyzed polymorphisms were selected for current study based on their association with tendinopathy. In athletes suffering from tendinopathy, carriage of the T allele (rs591058) was associated with a higher risk of developing disease manifestation episodes [17], while the C allele was associated with higher risk of anterior cruciate ligament rupture [31]. Other research has shown a relationship between the rs591058 CC genotype as well as the CC genotype of the rs679620 and a higher risk of Achilles tendinopathy (AT) [15]. Allele C of rs679620 was also associated with increased AT risk in another study [16], while the T allele was shown to reduce Achilles tendon rupture risk [32]. Although there are some discrepancies in the literature for rs591058, there is a visible association between the rs679620 C allele and an increased risk of tendon injuries. This is consistent with the results obtained in the current study. In our study, the C allele was associated with more frequent pain even before treatment, and reduced *MMP3* gene expression. The rs679620 polymorphism leads to a Lys-Glu substitution in the 45th amino acid of MMP3 [24,33]. However, this substitution does not affect the enzymatic activity of the protein [33,34]. There are circumstantial reports in the literature suggesting an association of the C allele with increased gene expression, although these are not proven assumptions [35]. Based on the presented information, it seems that rs679620 has a potential modulatory role in the functioning of MMP3, which is reflected in the clinical phenotype (for example, by increasing the risk of tendinopathy), but the exact mechanism of action is not known and requires further research.

In the case of the rs520540 polymorphism, there are no reports in the literature regarding its association with the risk of tendinopathy. However, this polymorphism showed a relationship with the risk of osteoarthritis in a male population from northern China [36]. In addition, rs520540 has also been associated with early periodontitis [37] and cerebral stroke risk [38]. Although this polymorphism was not shown to be associated with tendinopathy, it is important to emphasize that it occurs in strong linkage disequilibrium with the other SNPs presented in the current study. This suggests that such an association may exist, even though it has not been investigated to date.

As mentioned, MMP3 is present in healthy tendons [9], it is responsible for degradation of collagen (among other proteins) and participates in tissue remodeling [13]. Importantly, *MMP3* expression levels are reduced in tendinopathic or ruptured tendons [14,39,40]. Moreover, studies of supraspinatus and biceps tendons have shown that MMP3 levels are higher in the former, where collagen turnover is greater. Furthermore, ruptured supraspinatus tendons exhibit reduced *MMP3* expression and a deterioration of the collagen network [41]. This suggests that MMP3 has a significant role in tendon repair and/or maintenance [9], and its reduced expression during tendinopathy may disrupt these processes. Interestingly, in the current study, genotypes potentially associated with lower expression levels are also associated with more frequent pain (GG rs520540, CC rs591058, and CC rs679620). The same genotypes are also related to a higher risk of tendinopathy, which may be caused by reduced MMP3 remodeling activity.

Interestingly, PRP has been shown to reduce MMP3 levels. This relationship was demonstrated in osteoarthritic chondrocytes co-cultured with various PRP concentrations (the decrease in MMP3 was dose-dependent) [42], and in fibroblast-like synoviocytes from osteoarthritic knees (only in combination with interleukin-1 beta (IL-1β)) [43]. In contrast, another study of fibroblast-like synoviocytes treated with PRP (without the addition of IL-1β) showed an increase in MMP3 levels [44]. Furthermore, PRP contains and secretes MMP3, a significant portion of which is in its active form (depending on platelet count). It also induces the release of MMPs by ligament fibroblasts (dependent on prior IL-1β stimulation) [45]. This can have both positive and negative effects on tissue regeneration during tendinopathy. Delivering MMP3 via PRP to tissues with reduced levels of this protein may enable a return to proper concentration and positively impact tissue remodeling. On the other hand, excessively high MMPs levels can lead to increased tissue degradation. Based on the studies available in the literature, it is difficult to clearly determine how PRP affects the levels and secretion of MMP3 by damaged tissues. This process probably depends on the dose, platelet count, presence of interleukins, type of tissue (the studies cited do not include tenocytes), and many other factors. In our study, we linked alleles potentially associated with lower levels of gene expression (G rs520540, C rs591058, and C rs679620) with improved treatment effectiveness. This suggests a better therapeutic outcome resulting from lower MMP levels and reduced degenerative properties. As mentioned, MMPs may be associated with both healing and the pathogenesis of tendinopathy [7,8,9,10]; therefore, their levels must be properly controlled. Both too low and too high MMP levels can negatively impact the treatment of tendinopathy. In addition, the administration of PRP alone can potentially affect MMP3 levels, which adds an additional layer of complication to the entire process. It should be emphasized, however, that the association between *MMP3* gene polymorphisms and PRP therapy effectiveness in this study is weak (few statistically significant points in numerous analyses).

The main limitations of this study include the lack of a post-injection rehabilitation protocol and the small study group. Patients were not forbidden to receive other forms of treatment after PRP therapy, which may affect results. In our opinion, however, limiting treatment options for patients who have not achieved the desired effects of PRP therapy over a two-year period would be unethical. Moreover, we attempted to compensate for the small number of patients in the study group by using both quantitative and qualitative statistical tests, along with appropriate corrections for multiple testing, to minimize the risk of false positive results. Furthermore, power analyses indicated that the obtained study group may be sufficient. To achieve a power of 0.9 for the PRTEE analyses at week 104, the minimum number of cases was approximately 60. Importantly, other potentially confounding factors (such as age, gender, BMI) can also influence the obtained results. Additionally, in the case of the *MMP3* gene, a significant factor potentially influencing the results is the strong linkage disequilibrium between the studied polymorphisms. However, it should be emphasized that, in the present study, the haplotype analyses mostly overlap with individual polymorphism analyses. Further research on a larger study population is required to confirm our findings.

## 4. Materials and Methods

### 4.1. Study Design

This prospective cohort study included patients diagnosed with lateral elbow tendinopathy (tennis elbow) treated with PRP injection. The study was conducted in accordance with the STROBE and MIBO guidelines as well as the principles of the 1975 Declaration of Helsinki and its subsequent amendments. The research protocol received approval from the Ethics Committee of the Medical University of Silesia in Katowice, Poland (KNW/0022/KB1/24/I/17). Written informed consent was obtained from all study participants.

The methodology followed identical effectiveness measures, follow-up schedule, patient selection criteria, PRP preparation and injection procedures, as well as blood analyses as in our previous studies [46]. Patients were followed for 104 weeks, with assessments at 2, 4, 8, 12, 24, 52, and 104 weeks post-injection. The effectiveness of therapy was evaluated using the patient-reported outcome measures (PROMs), namely, visual analogue scale (VAS), quick version of the disabilities of the arm, shoulder and hand (QDASH), and the patient-rated tennis elbow evaluation (PRTEE) scale. Minimal clinically important difference (MCID) thresholds were set at 1.5 points for VAS [47], 15.8 points for QDASH [48], and 11 points for PRTEE [49]. Patients who achieved the MCID at the follow-up were classified as responders, while those who did not were classified as non-responders. Genetic testing was performed for selected SNPs in the *MMP3* gene and the clinical phenotype and efficacy of PRP injection were compared between genotypic variants of SNPs.

### 4.2. Patient Selection and Characteristics

Between November 2018 and November 2019, 107 Polish Caucasian patients (132 elbows, including 25 bilateral cases) with lateral elbow tendinopathy (LET, ICD-10: M77.1) were enrolled in this study at two centers: the VI Department of Trauma and Orthopaedics, District Hospital of Orthopedics and Trauma Surgery in Piekary Śląskie, and the Department of Orthopedic Trauma Surgery, Multidisciplinary Hospital in Jaworzno. Follow-up data were collected until November 2021. All patients were examined, qualified, and injected according to an identical study protocol.

The study group consisted of 65 women and 42 men aged 24–64 years (median ± QD: 46.00 ± 5.50). The most common comorbidities were hypertension, thyroid disorders, and gout. Blood morphology at baseline showed an average WBC count of 6.26 ± 1.16 × 10^9^/L, platelet count of 240.00 ± 40.50 × 10^9^/L, and MPV of 9.10 ± 0.73 fL. Women had significantly higher platelet counts (261.50 ± 33.00 vs. 224.00 ± 38.75, *p* = 0.000) and plateletcrit values (2.37 ± 0.36 vs. 2.04 ± 0.33, *p* = 0.001) compared with men. No significant sex-related differences were found in PRP platelet parameters. Before PRP administration (day 0), pain characteristics were assessed, including pain radiation from the lateral epicondyle of the humerus (to the wrist, forearm, or neck) and pain exacerbated by specific activities (holding, lifting, or grasping objects). Patients most often experienced pain during lifting, which radiated primarily to the forearm. Women experience wrist pain (38.90% women vs. 18.18% men, *p* = 0.018) and shoulder pain (24.68% women vs. 7.27% men, *p* = 0.018) more often than men. A summary of key demographic and clinical data is provided in Table 3.

The inclusion and exclusion criteria were similar to those in previously published papers [46]. Inclusion criteria were symptoms typical of LET persisting for ≥3 months, including pain at the common extensor origin, grip strength weakness, morning stiffness, tenderness over the lateral epicondyle, and positive Thomson’s, Mill’s, and Cozen’s tests. Exclusion criteria were other injuries or diseases of the affected limb, rheumatoid arthritis, malignancy, cervical radiculopathy, pregnancy, prior surgery or PRP injection, local corticosteroid injection within 6 months, use of antiplatelet medication, or cognitive impairment.

**Figure 5:**
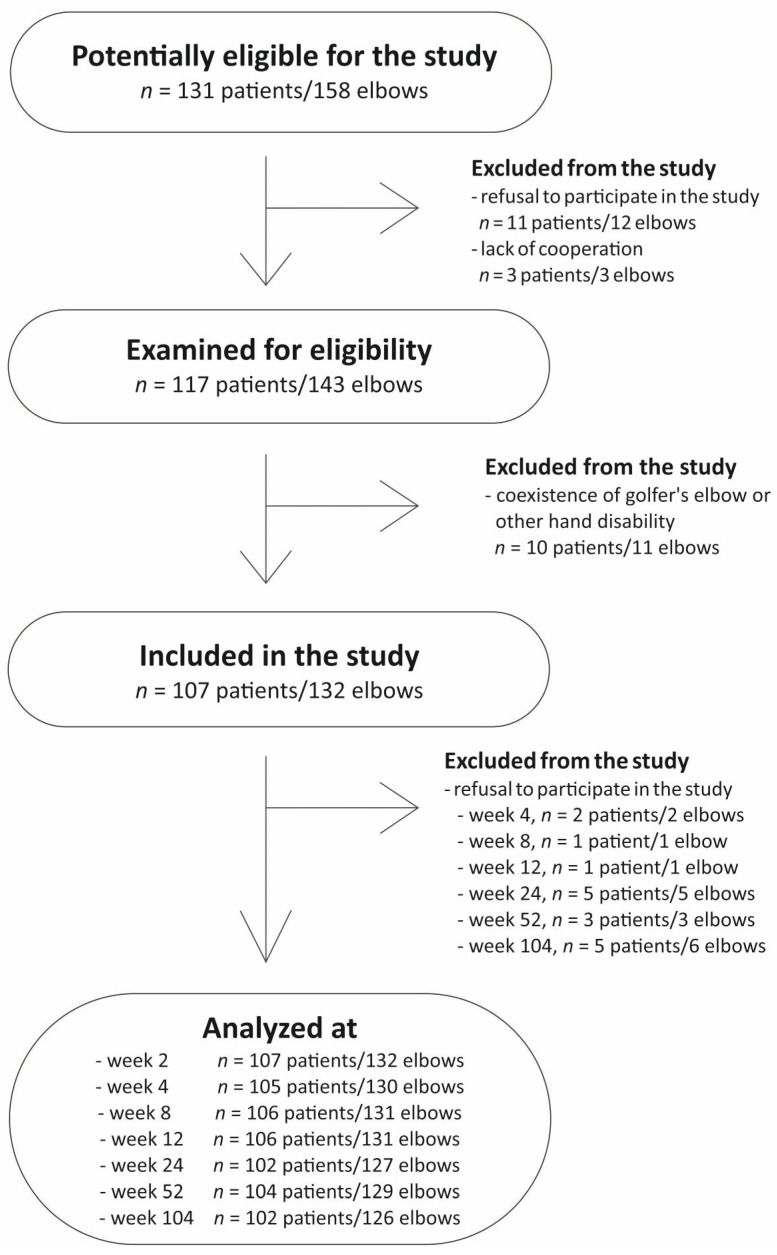
Flowchart of the study selection.

There was no standardized post-injection rehabilitation protocol. Additional therapies during follow-up (physiotherapy, NSAIDs, corticosteroids, or further PRP injections) were monitored but not considered exclusion criteria. A flow diagram of patient selection is shown in Figure 5.

### 4.3. PRP Separation and Injection Procedure

For each patient, 12 mL of fresh whole blood was collected under standardized conditions (20 °C, identical light exposure) using disposable materials and an Arthrex Autologous Conditioned Plasma (ACP) double syringe (Arthrex GmbH, München, Germany). The blood was immediately mixed with 3.13% sodium citrate (MediPac^®^ GmbH, Königswinter, Germany) at a 9:1 ratio and centrifuged in a Rotofix 32A centrifuge (Andreas Hettich GmbH & Co., Tuttlingen, Germany) at 1500 rpm for 5 min.

This process yielded 2.5–3.5 mL of PRP, of which 2.0–3.0 mL was injected directly into the region of the common extensor origin under ultrasound guidance (Mindray DC-3 device, equipped with a 5–10 MHz linear probe) using a 1.2 mm needle. In bilateral cases, blood was collected and PRP was prepared separately for each elbow. The remaining ~0.5 mL of PRP was preserved for laboratory analyses.

All injections were performed by two senior orthopedic surgeons (K.S. in Piekary Śląskie and W.K. in Jaworzno) with over 17–20 years of clinical experience, following an identical study protocol. Patients were monitored for 30 min post-injection (particularly local inflammation and allergic reactions) and advised to avoid heavy limb use for 24 h. No infections or serious complications were observed.

### 4.4. Genetic Analyses

Peripheral blood samples were collected on the day of PRP injection. Genomic DNA was isolated from peripheral blood leukocytes using the MasterPure DNA purification kit (Epicenter Technologies, Madison, WI, USA).

**Figure 6:**
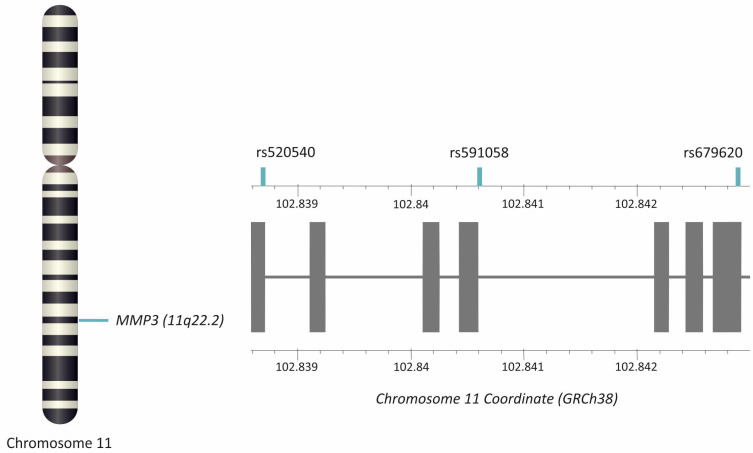
Location of the *MMP3* gene and studied polymorphisms. Gray squares represent exons. The figure was created using the LD-Matrix tool [50]. Legend: MMP3, matrix metalloproteinase 3.

Three selected *MMP3* gene polymorphisms were analyzed: rs520540 (A > G), rs591058 (T > C), and rs679620 (T > C) (Figure 6). The choice of variants was based on the National Center for Biotechnology Information (NCBI) dbSNP database (U.S. National Library of Medicine) [24], with the criterion that the minor allele frequency (MAF) in European populations was ≥20%. Furthermore, selected SNPs show an association with the clinical phenotype [15,16,17,36].

Genotyping was performed using TaqMan Predesigned SNP Genotyping Assay kits (Thermo Fisher Scientific, Waltham, MA, USA) and the LightCycler^®^ 480 Real-Time PCR System (F. Hoffmann-La Roche AG, Basel, Switzerland). To ensure reliability, 10–15% of randomly selected samples were regenotyped, and the results showed 100% repeatability.

### 4.5. Statistical Analysis

Statistical analyses were performed using Statistica 13.0 software (TIBCO Software Inc., Palo Alto, CA, USA). The normality of quantitative variables was assessed using the Shapiro–Wilk test. Since all quantitative variables displayed non-normal distributions, data were reported as medians with interquartile range (QD). Analyses were performed for 132 cases (132 elbows). Cases with missing data were excluded from relevant analyses.

Genetic data were analyzed under dominant/recessive and additive inheritance models. Differences in patient-reported outcome measures (VAS, QDASH, PRTEE) and their changes from baseline (ΔVAS, ΔQDASH, ΔPRTEE) were compared between carriers of different genotypes using the non-parametric Mann–Whitney U test. Hardy–Weinberg equilibrium was tested using the χ^2^ test. Genotype and allele frequencies were compared between categories of qualitative variables (achievement of MCID, pain before therapy) using the χ^2^ test; Yates’ correction was applied for subgroups with fewer than ten subjects. In silico analyses of expression levels were performed using the GTEx tool version V10 [25].

Haplotype blocks were determined using HaploView 4.2 software (Broad Institute of MIT and Harvard, Cambridge, MA, USA) [51] with the Gabriel et al. algorithm [52]. Linkage disequilibrium was assessed using D′ and R^2^ values.

Sex-based comparisons of blood morphological parameters between genders were made using the Mann–Whitney U test. Power analyses were performed using the Student’s *t*-test for independent groups. Relative risk (RR) was calculated using the Epi Info^TM^ version 7.2.2.16 tool [53]. The Cohen’s *d* value was calculated according to the formula (median_1_ − median_2_)/QD. Haplotype analyses were performed using logistic regression for carriers vs. non-carriers of the GCC haplotype. Statistical significance was set at *p* < 0.05. Multiple comparisons were accounted for using the Hochberg correction (for related hypotheses) [54].

## 5. Conclusions

In the current study, we found only a weak association between the studied polymorphisms and treatment efficacy. This indicates that, although *MMP3* is associated with the pathogenesis of tendinopathy, and polymorphisms in this gene are associated with the risk of its occurrence, this does not translate into an association with the effectiveness of tendinopathy treatment using platelet-rich plasma. Nevertheless, the analyzed polymorphisms were associated with pain experienced by patients before treatment, which may result from the association of MMP3 with tissue remodeling and reduced *MMP3* expression in carriers of specific genotypes.

## Figures and Tables

**Table 1:** The studied polymorphisms of the *MMP3* gene—genotypes and alleles frequencies, chromosomal location, Hardy–Weinberg equilibrium *p* value.

*MMP3* SNPs	Chromosomal Localization *	Alleles	*n* **	%	Genotypes	*n* **	%	HWE *p* Value
rs520540	11:102,838,694	A	97	48.99	AA	31	23.48	0.999
		G	101	51.01	AG	66	50.00	
					GG	35	26.52	
rs591058	11:102,840,607	T	96	48.73	TT	31	23.48	0.988
		C	101	51.27	TC	65	49.24	
					CC	36	27.27	
rs679620	11:102,842,889	T	96	48.73	TT	31	23.48	0.988
		C	101	51.27	TC	65	49.24	
					CC	36	27.27	

Legend: SNP, single nucleotide polymorphism; HWE, Hardy–Weinberg equilibrium; *, GRCh38. **—data for 132 cases (132 elbows).

**Table 2:** The distribution of genotype frequencies of the studied polymorphisms in additive and dominant/recessive models for patients who achieved MCID threshold (responders) and who did not (non-responders) after PRP therapy (PRTEE week 104).

Model of Heredity	SNP	Genotype	Responders		Non-Responders		RR (95% CI)	*p*
			*n*	%	*n*	%		
Additive	rs520540	AA	20	18.87	9	52.94		0.007
		AG	53	50.00	6	35.29	0.52 (0.29–0.93)	
		GG	33	31.13	2	11.76		
	rs591058/rs679620	TT	20	18.87	9	52.94		0.017
		TC	52	49.06	6	35.29	0.52 (0.30–0.94)	
		CC	34	32.08	2	11.76		
Dominant/recessive	rs520540	AA	20	18.87	9	52.94		0.006
		AG/GG	86	81.13	8	47.06		
	rs591058/rs679620	TT	20	18.87	9	52.94		0.006
		TC/CC	86	81.13	8	47.06		

Legend: SNP, single nucleotide polymorphism; PROM, patient-reported outcome measures; MCID, minimal clinically important difference; PRTEE, patient-rated tennis elbow evaluation; RR, relative risk; CI, confidence interval.

**Table 3:** Clinical characteristics of the studied group.

Characteristics			
General	number of subjects, *N*	107	-
	number of elbows, *n* (%)	132	(100.00)
	age, median ± QD	46.00	5.50
	BMI, median ± QD	25.65	2.00
	current smokers, *n* (%)	22	(16.67)
Comorbidities	diabetes mellitus, *n* (%)	4	(3.03)
	gout, *n* (%)	8	(6.06)
	obesity (BMI ≥ 30), *n* (%)	26	(19.70)
	overweight/obesity (BMI ≥ 25), *n* (%)	86	(65.15)
	hypercholesterolemia, *n* (%)	10	(7.58)
	hypertension, *n* (%)	18	(13.64)
Pain of elbow	in LE area, *n* (%)	132	(100.00)
	during lifting, *n* (%) *	117	(90.00)
	when grabbing, *n* (%) *	80	(61.54)
	when pressing, *n* (%) *	85	(65.39)
Pain radiating to the	wrist, *n* (%) *	40	(30.77)
	forearm, *n* (%) *	65	(50.00)
	neck, *n* (%) *	12	(9.23)

Legend: QD, quartile deviation; BMI, body mass index; LE, lateral epicondyle of the humerus; * data available for 130 elbows.

## Data Availability

The original contributions presented in this study are included in this article/Supplementary Materials; further inquiries can be directed to the corresponding authors.

## Supplementary Materials

The following supporting information can be downloaded at: https://www.mdpi.com/article/10.3390/ijms262110579/s1.
