# Supplementary material for: The Association Between Three MMP3 Gene Polymorphisms and the Efficacy of Platelet-Rich Plasma Therapy in the Treatment of Lateral Elbow Tendinopathy—A Prospective Cohort Study
Source: Int J Mol Sci. 2025 Oct 30;26(21):10579. doi: 10.3390/ijms262110579 (PMC12610566; doi:10.3390/ijms262110579)
Supplement: Supplementary file 1 [file ijms-26-10579-s001.zip › ijms-3938527-supplementary.pdf]

Table S1. PROMs values (median ± QD) in carriers of different genotypes of the rs520540 polymorphism of the *MMP3* gene in subsequent weeks of follow-up (dominant/recessive model).

PROMs values in AA homozygotes and G allele carriers of the rs520540 *MMP3* gene polymorphism.

**PROMs**		**AA rs520540**		**AG+GG rs520540**		***p***
	week	median	± QD	median	± QD	**Mann-Whitney U test**
VAS	0	6.00	1.50	6.00	2.00	0.856
	2	3.00	1.50	4.00	1.50	0.198
	4	2.50	1.50	3.00	1.50	**0.047**
	8	2.00	1.50	3.00	2.00	0.105
	12	2.00	1.50	3.00	2.00	0.096
	24	1.00	1.50	2.00	2.00	0.283
	52	1.00	2.00	2.00	2.00	0.704
	104	2.00	2.00	1.00	1.50	0.269
ΔVAS (vs week 0)	2	1.00	2.00	1.00	1.50	0.340
	4	3.00	1.50	2.00	2.00	0.098
	8	3.00	2.00	2.00	2.00	0.230
	12	3.50	2.50	2.00	2.25	0.144
	24	4.00	2.50	3.00	1.50	0.324
	52	3.50	3.00	3.50	2.00	0.820
	104	3.00	2.50	4.00	2.00	0.429
QDASH	0	52.27	14.77	52.27	12.50	0.789
	2	37.50	14.77	40.91	15.91	0.483
	4	32.95	12.50	36.36	14.77	0.341
	8	27.27	18.18	34.09	17.05	0.152
	12	25.00	19.32	29.55	17.05	0.138
	24	14.77	20.45	25.00	21.59	0.360
	52	20.45	25.00	18.18	21.59	0.798
	104	27.27	22.73	11.36	14.78	0.261
ΔQDASH (vs week 0)	2	9.09	19.41	6.81	13.14	0.798
	4	17.72	11.36	10.22	15.91	0.392
	8	20.45	17.04	12.50	18.75	0.203
	12	29.45	20.46	17.04	17.05	0.183
	24	31.81	22.73	18.17	18.05	0.364
	52	20.45	26.14	22.73	19.32	0.967
	104	22.72	26.14	34.09	21.59	0.109
PRTEE	0	50.00	13.00	52.50	14.25	0.336
	2	25.50	12.50	32.50	16.75	0.375
	4	20.50	12.75	26.50	14.50	0.078
	8	18.50	11.50	24.00	17.75	0.068
	12	15.50	11.50	21.50	17.25	0.063
	24	9.50	12.50	15.75	17.50	0.104
	52	11.50	12.75	12.00	15.75	0.463
	104	16.50	16.25	7.00	9.75	0.230
ΔPRTEE (vs week 0)	2	11.75	14.00	15.25	11.50	0.715
	4	23.75	13.50	21.25	13.38	0.566
	8	28.00	14.50	25.50	17.00	0.482
	12	29.50	15.75	28.50	16.38	0.439
	24	34.50	18.50	28.50	15.75	0.414
	52	29.00	20.00	33.50	16.50	0.740
	104	25.00	22.00	38.75	12.75	**0.023***

PROMs values in GG homozygotes and A allele carriers of the rs520540 *MMP3* gene polymorphism.

PROMs	week	GG rs520540 median	GG rs520540 ± QD	AG+AA rs520540 median	AG+AA rs520540 ± QD	*p* Mann-Whitney U test
VAS	0	6.00	1.75	6.00	6.00	0.311
	2	3.00	1.50	4.00	4.00	0.810
	4	3.00	1.50	3.00	3.00	0.957
	8	3.00	2.50	3.00	3.00	0.723
	12	2.00	2.00	3.00	3.00	0.586
	24	2.00	2.50	2.00	2.00	0.533
	52	1.00	2.50	2.00	2.00	0.867
	104	1.00	1.00	1.00	1.00	0.223
ΔVAS (vs week 0)	2	2.00	1.50	1.00	1.00	0.471
	4	2.00	1.50	2.00	2.00	0.480
	8	3.00	2.50	2.00	2.00	0.875
	12	2.00	2.50	3.00	3.00	0.903
	24	2.00	2.50	3.00	3.00	0.968
	52	4.00	2.50	3.00	3.00	0.349
	104	4.00	2.50	4.00	4.00	0.134
QDASH	0	52.27	9.09	52.27	52.27	0.162
	2	43.18	20.45	38.64	38.64	0.486
	4	34.09	18.18	36.36	36.36	0.907
	8	34.09	19.32	31.82	31.82	0.712
	12	29.55	21.59	27.27	27.27	0.350
	24	25.00	23.86	25.00	25.00	0.547
	52	15.91	21.59	18.18	18.18	0.875
	104	15.91	17.05	13.64	13.64	0.925
ΔQDASH (vs week 0)	2	11.36	18.18	6.81	6.81	0.843
	4	18.17	18.18	11.36	11.36	0.288
	8	20.45	18.19	14.77	14.77	0.571
	12	20.45	15.91	18.18	18.18	0.948
	24	18.18	17.05	20.45	20.45	0.689
	52	31.82	17.95	19.31	19.31	0.187
	104	38.63	23.87	29.50	29.50	0.201
PRTEE	0	55.00	13.50	51.00	51.00	0.133
	2	34.50	16.00	28.50	28.50	0.592
	4	27.50	16.25	24.50	24.50	0.965
	8	22.00	19.25	22.00	22.00	0.708
	12	23.00	23.75	19.50	19.50	0.438
	24	14.00	20.50	15.00	15.00	0.606
	52	13.00	14.00	11.50	11.50	0.722
	104	8.00	9.75	7.00	7.00	0.943
ΔPRTEE (vs week 0)	2	21.00	14.75	14.00	14.00	0.409
	4	26.50	13.00	20.50	20.50	0.233
	8	32.00	15.95	24.75	24.75	0.473
	12	29.50	15.20	26.75	26.75	0.981
	24	31.50	18.00	30.50	30.50	0.824
	52	40.00	15.50	30.00	30.00	0.170
	104	40.00	10.25	37.00	37.00	0.104

Legend: *MMP3*, matrix metalloproteinase 3; QD, quartile deviation; VAS, visual analog scale; QDASH, quick version of disabilities of the arm, shoulder and hand score; PRTEE, patient-rated tennis elbow evaluation; PROM, patient-reported outcome measures. *statistically significant after Hochberg correction ($p \leq 0.023$).

Table S2. PROMs values (median ± QD) in carriers of different genotypes of the rs591058 polymorphism of the *MMP3* gene in subsequent weeks of follow-up (dominant/recessive model).

PROMs values in TT homozygotes and C allele carriers of the rs591058 *MMP3* gene polymorphism.

**PROMs**		**TT rs591058**		**TC+CC rs591058**		***p***
	week	median	± QD	median	± QD	**Mann-Whitney U test**
VAS	0	6.00	1.50	6.00	2.00	0.856
	2	3.00	1.50	4.00	1.50	0.198
	4	2.50	1.50	3.00	1.50	**0.047**
	8	2.00	1.50	3.00	2.00	0.105
	12	2.00	1.50	3.00	2.00	0.096
	24	1.00	1.50	2.00	2.00	0.283
	52	1.00	2.00	2.00	2.00	0.704
	104	2.00	2.00	1.00	1.50	0.269
ΔVAS (vs week 0)	2	1.00	2.00	1.00	1.50	0.340
	4	3.00	1.50	2.00	2.00	0.098
	8	3.00	2.00	2.00	2.00	0.230
	12	3.50	2.50	2.00	2.25	0.144
	24	4.00	2.50	3.00	1.50	0.324
	52	3.50	3.00	3.50	2.00	0.820
	104	3.00	2.50	4.00	2.00	0.429
QDASH	0	52.27	14.77	52.27	12.50	0.789
	2	37.50	14.77	40.91	15.91	0.483
	4	32.95	12.50	36.36	14.77	0.341
	8	27.27	18.18	34.09	17.05	0.152
	12	25.00	19.32	29.55	17.05	0.138
	24	14.77	20.45	25.00	21.59	0.360
	52	20.45	25.00	18.18	21.59	0.798
	104	27.27	22.73	11.36	14.78	0.261
ΔQDASH (vs week 0)	2	9.09	19.41	6.81	13.14	0.798
	4	17.72	11.36	10.22	15.91	0.392
	8	20.45	17.04	12.50	18.75	0.203
	12	29.45	20.46	17.04	17.05	0.183
	24	31.81	22.73	18.17	18.05	0.364
	52	20.45	26.14	22.73	19.32	0.967
	104	22.72	26.14	34.09	21.59	0.109
PRTEE	0	50.00	13.00	52.50	14.25	0.336
	2	25.50	12.50	32.50	16.75	0.375
	4	20.50	12.75	26.50	14.50	0.078
	8	18.50	11.50	24.00	17.75	0.068
	12	15.50	11.50	21.50	17.25	0.063
	24	9.50	12.50	15.75	17.50	0.104
	52	11.50	12.75	12.00	15.75	0.463
	104	16.50	16.25	7.00	9.75	0.230
ΔPRTEE (vs week 0)	2	11.75	14.00	15.25	11.50	0.715
	4	23.75	13.50	21.25	13.38	0.566
	8	28.00	14.50	25.50	17.00	0.482
	12	29.50	15.75	28.50	16.38	0.439
	24	34.50	18.50	28.50	15.75	0.414
	52	29.00	20.00	33.50	16.50	0.740
	104	25.00	22.00	38.75	12.75	**0.023***

PROMs values in CC homozygotes and T allele carriers of the rs591058 *MMP3* gene polymorphism.

PROMs	week	CC rs591058 median	CC rs591058 ± QD	TC+CC rs591058 median	TC+CC rs591058 ± QD	p Mann-Whitney U test
VAS	0	6.00	1.88	6.00	1.50	0.468
	2	3.00	1.50	4.00	1.00	0.672
	4	3.00	1.75	3.00	1.50	0.776
	8	3.00	2.25	3.00	1.75	0.885
	12	2.00	2.00	3.00	1.50	0.701
	24	2.00	2.25	2.00	1.75	0.618
	52	1.00	2.25	2.00	2.00	0.822
	104	1.00	1.00	1.00	1.50	0.160
ΔVAS (vs week 0)	2	1.75	1.50	1.00	1.50	0.595
	4	2.00	1.50	2.00	2.00	0.560
	8	2.50	2.50	2.00	2.00	0.957
	12	2.00	2.25	3.00	2.00	0.792
	24	2.00	2.50	3.00	2.00	0.838
	52	4.00	2.50	3.00	2.00	0.431
	104	4.00	2.50	4.00	2.50	0.180
QDASH	0	52.27	9.09	52.27	13.98	0.293
	2	42.05	19.89	38.64	13.64	0.714
	4	31.82	17.05	36.36	12.50	0.672
	8	32.95	18.75	32.95	19.32	0.945
	12	28.41	22.16	28.41	15.91	0.484
	24	22.73	23.86	25.00	20.45	0.694
	52	14.77	21.02	19.32	23.86	0.920
	104	14.77	14.77	13.64	21.59	0.740
ΔQDASH (vs week 0)	2	10.22	17.61	6.81	11.36	0.928
	4	15.90	17.61	11.36	13.64	0.368
	8	19.32	18.19	15.90	20.45	0.687
	12	20.45	16.48	18.18	18.18	0.884
	24	17.05	17.55	20.45	20.46	0.833
	52	31.82	17.50	20.45	22.73	0.264
	104	38.63	23.30	29.54	22.73	0.296
PRTEE	0	55.00	13.63	51.25	13.38	0.244
	2	34.25	16.38	29.00	16.75	0.811
	4	25.25	16.50	24.50	12.75	0.823
	8	22.00	18.63	23.00	14.63	0.901
	12	22.25	22.25	19.75	14.13	0.540
	24	13.75	19.50	15.00	16.75	0.683
	52	12.00	13.63	11.75	15.50	0.850
	104	7.75	9.00	7.00	14.38	0.761
ΔPRTEE (vs week 0)	2	19.75	14.25	14.00	11.25	0.509
	4	26.45	13.13	20.75	13.25	0.311
	8	31.75	15.85	25.00	16.75	0.585
	12	29.25	15.85	27.00	17.25	0.855
	24	30.00	17.00	30.50	19.25	0.994
	52	40.00	15.38	31.00	17.75	0.244
	104	39.75	10.50	37.50	18.50	0.163

Legend: *MMP3*, matrix metalloproteinase 3; QD, quartile deviation; VAS, visual analog scale; QDASH, quick version of disabilities of the arm, shoulder and hand score; PRTEE, patient-rated tennis elbow evaluation; PROM, patient-reported outcome measures. *statistically significant after Hochberg correction ($p \leq 0.023$).

Table S3. PROMs values (median ± QD) in carriers of different genotypes of the rs679620 polymorphism of the *MMP3* gene in subsequent weeks of follow-up (dominant/recessive model).

PROMs values in TT homozygotes and C allele carriers of the rs679620 *MMP3* gene polymorphism.

**PROMs**		**TT rs679620**		**TC+CC rs679620**		***p***
	week	median	± QD	median	± QD	**Mann-Whitney U test**
VAS	0	6.00	1.50	6.00	2.00	0.856
	2	3.00	1.50	4.00	1.50	0.198
	4	2.50	1.50	3.00	1.50	**0.047**
	8	2.00	1.50	3.00	2.00	0.105
	12	2.00	1.50	3.00	2.00	0.096
	24	1.00	1.50	2.00	2.00	0.283
	52	1.00	2.00	2.00	2.00	0.704
	104	2.00	2.00	1.00	1.50	0.269
ΔVAS (vs week 0)	2	1.00	2.00	1.00	1.50	0.340
	4	3.00	1.50	2.00	2.00	0.098
	8	3.00	2.00	2.00	2.00	0.230
	12	3.50	2.50	2.00	2.25	0.144
	24	4.00	2.50	3.00	1.50	0.324
	52	3.50	3.00	3.50	2.00	0.820
	104	3.00	2.50	4.00	2.00	0.429
QDASH	0	52.27	14.77	52.27	12.50	0.789
	2	37.50	14.77	40.91	15.91	0.483
	4	32.95	12.50	36.36	14.77	0.341
	8	27.27	18.18	34.09	17.05	0.152
	12	25.00	19.32	29.55	17.05	0.138
	24	14.77	20.45	25.00	21.59	0.360
	52	20.45	25.00	18.18	21.59	0.798
	104	27.27	22.73	11.36	14.78	0.261
ΔQDASH (vs week 0)	2	9.09	19.41	6.81	13.14	0.798
	4	17.72	11.36	10.22	15.91	0.392
	8	20.45	17.04	12.50	18.75	0.203
	12	29.45	20.46	17.04	17.05	0.183
	24	31.81	22.73	18.17	18.05	0.364
	52	20.45	26.14	22.73	19.32	0.967
	104	22.72	26.14	34.09	21.59	0.109
PRTEE	0	50.00	13.00	52.50	14.25	0.336
	2	25.50	12.50	32.50	16.75	0.375
	4	20.50	12.75	26.50	14.50	0.078
	8	18.50	11.50	24.00	17.75	0.068
	12	15.50	11.50	21.50	17.25	0.063
	24	9.50	12.50	15.75	17.50	0.104
	52	11.50	12.75	12.00	15.75	0.463
	104	16.50	16.25	7.00	9.75	0.230
ΔPRTEE (vs week 0)	2	11.75	14.00	15.25	11.50	0.715
	4	23.75	13.50	21.25	13.38	0.566
	8	28.00	14.50	25.50	17.00	0.482
	12	29.50	15.75	28.50	16.38	0.439
	24	34.50	18.50	28.50	15.75	0.414
	52	29.00	20.00	33.50	16.50	0.740
	104	25.00	22.00	38.75	12.75	**0.023***

PROMs values in CC homozygotes and T allele carriers of the rs679620 *MMP3* gene polymorphism.

PROMs	week	CC rs679620 median	CC rs679620 ± QD	TC+CC rs679620 median	TC+CC rs679620 ± QD	*p* Mann-Whitney U test
VAS	0	6.00	1.88	6.00	1.50	0.468
	2	3.00	1.50	4.00	1.00	0.672
	4	3.00	1.75	3.00	1.50	0.776
	8	3.00	2.25	3.00	1.75	0.885
	12	2.00	2.00	3.00	1.50	0.701
	24	2.00	2.25	2.00	1.75	0.618
	52	1.00	2.25	2.00	2.00	0.822
	104	1.00	1.00	1.00	1.50	0.160
ΔVAS (vs week 0)	2	1.75	1.50	1.00	1.50	0.595
	4	2.00	1.50	2.00	2.00	0.560
	8	2.50	2.50	2.00	2.00	0.957
	12	2.00	2.25	3.00	2.00	0.792
	24	2.00	2.50	3.00	2.00	0.838
	52	4.00	2.50	3.00	2.00	0.431
	104	4.00	2.50	4.00	2.50	0.180
QDASH	0	52.27	9.09	52.27	13.98	0.293
	2	42.05	19.89	38.64	13.64	0.714
	4	31.82	17.05	36.36	12.50	0.672
	8	32.95	18.75	32.95	19.32	0.945
	12	28.41	22.16	28.41	15.91	0.484
	24	22.73	23.86	25.00	20.45	0.694
	52	14.77	21.02	19.32	23.86	0.920
	104	14.77	14.77	13.64	21.59	0.740
ΔQDASH (vs week 0)	2	10.22	17.61	6.81	11.36	0.928
	4	15.90	17.61	11.36	13.64	0.368
	8	19.32	18.19	15.90	20.45	0.687
	12	20.45	16.48	18.18	18.18	0.884
	24	17.05	17.55	20.45	20.46	0.833
	52	31.82	17.50	20.45	22.73	0.264
	104	38.63	23.30	29.54	22.73	0.296
PRTEE	0	55.00	13.63	51.25	13.38	0.244
	2	34.25	16.38	29.00	16.75	0.811
	4	25.25	16.50	24.50	12.75	0.823
	8	22.00	18.63	23.00	14.63	0.901
	12	22.25	22.25	19.75	14.13	0.540
	24	13.75	19.50	15.00	16.75	0.683
	52	12.00	13.63	11.75	15.50	0.850
	104	7.75	9.00	7.00	14.38	0.761
ΔPRTEE (vs week 0)	2	19.75	14.25	14.00	11.25	0.509
	4	26.45	13.13	20.75	13.25	0.311
	8	31.75	15.85	25.00	16.75	0.585
	12	29.25	15.85	27.00	17.25	0.855
	24	30.00	17.00	30.50	19.25	0.994
	52	40.00	15.38	31.00	17.75	0.244
	104	39.75	10.50	37.50	18.50	0.163

Legend: *MMP3*, matrix metalloproteinase 3; QD, quartile deviation; VAS, visual analog scale; QDASH, quick version of disabilities of the arm, shoulder and hand score; PRTEE, patient-rated tennis elbow evaluation; PROM, patient-reported outcome measures. *statistically significant after Hochberg correction ($p \leq 0.023$).

Table S4. The **distribution** of genotype frequencies of the *MMP3* gene polymorphisms for patients experiencing hand pain (in additive and dominant/recessive models).

Model of Heredity	SNP	Genotype	Presence of pain			No pain			*p* value
			n	genotype carriers [%]	patients [%]	n	genotype carriers [%]	patients [%]	
Additive	rs520540	AA	2	11.76	6.45	29	25.66	93.55	0.004
		AG	5	29.41	7.69	60	53.10	92.31	
		GG	10	58.82	29.41	24	21.24	70.59	
	rs591058	TT	2	11.76	6.45	29	25.66	93.55	0.006
		TC	5	29.41	7.81	59	52.21	92.19	
		CC	10	58.82	28.57	25	22.11	71.43	
	rs679620	TT	2	11.76	6.45	29	25.66	93.55	0.006
		TC	5	29.41	7.81	59	52.21	92.19	
		CC	10	58.82	28.57	25	22.11	71.43	
Dominant/recessive	rs520540	GG	10	58.82	51.72	24	21.24	48.28	0.003
		AG+AA	7	41.18	7.29	89	78.76	92.71	
	rs591058	CC	10	58.82	28.57	25	22.11	71.43	0.004
		TC+TT	7	41.18	7.37	88	77.88	92.63	
	rs679620	CC	10	58.82	28.57	25	22.11	71.43	0.004
		TC+TT	7	41.18	7.37	88	77.88	92.63	

Legend: SNP, single nucleotide polymorphisms; MMP3, matrix metalloproteinase 3. Hochberg correction *p* value assessed as 0.006).
